# Draft Genome Sequences and Genomic Analysis for Pigment Production in Bacteria Isolated from Blue Discolored Soymilk and Tofu

**DOI:** 10.7150/jgen.65500

**Published:** 2021-09-23

**Authors:** Marina E. De León, Harriet S. Wilson, Guillaume Jospin, Jonathan A. Eisen

**Affiliations:** 1 Department of Microbiology & Molecular Genetics, University of California, Davis, California 95616 USA.; 2 UC Davis Genome Center, 451 Health Science Drive, Davis, CA 95616 USA.; 3 Department of Biological Sciences, Sierra College, 5100 Sierra College Blvd, Rocklin, CA 95677 USA.; 4 Department of Evolution and Ecology, University of California, Davis, California 95616 USA.; 5 Department of Medical Microbiology and Immunology, University of California, Davis, California 95616 USA.; 6 AnimalBiome, Oakland, CA 94609 USA.

**Keywords:** Pseudomonas, Serratia, Soy, Blue discoloration, Pyomelanin, Food Contamination, Bacterial Pigment, Whole Genome Sequencing, 16S rRNA gene sequencing

## Abstract

Cold-tolerant bacteria are known to contaminate and cause defects in refrigerated foods. Defects in food products can be observed as changes in appearance, texture, and/or flavor that detract from the product's intended look, feel, or taste. Two distinct organisms were cultured from blue pigmented soymilk and tofu that had been left opened and expired in a home refrigerator. The blue coloration was reproduced when isolates were cultured in fresh, sterile soymilk. These strains also produced a variety of colony color morphologies when cultured on different media types. We report two draft genome sequences of the potential causative agents of blue discoloration of soy foods, *Pseudomonas carnis* strains UCD_MED3 and UCD_MED7 as well as the 16S rRNA gene sequences of co-occurring strains isolated from the defective soy samples but that did not cause blue discoloration when cultured in fresh soymilk; *Serratia liquefaciens* strains UCD_MED2 and UCD_MED5.

## Introduction

Information regarding how perishable foods, such as soy-based products, become contaminated and whether such contamination poses a possible health hazard can be significant aspects of food-borne illness prevention and food product waste prevention. Soy products are an inexpensive and healthy part of the human diet, and many people are using soy as a dietary staple in the United States 1, and throughout the world. Food defects such as blue discoloration of animal products used for human consumption including cheeses, milk and rabbit flesh have been reported in multiple case studies 2-4. Discoloration in such cases generally develops after a food package has been opened and a product has been exposed to air; also bacterial pigment production is usually reported to occur at lower temperatures but not higher temperatures 5. The causal agents for cases of blue discoloration have usually been identified as bacteria in the *Pseudomonas fluorescens* group. Prior phylogenetic studies have suggested that there is a “blue branch” (i.e. a clade) within the group where all the blue discoloration causing taxa can be found 6. Although many known blue-pigment producing strains are in the “blue branch”, this clade also includes unpigmented strains which suggests that the blue pigment production may involve core genes as opposed to accessory or peripheral genes in the *Pseudomonas* pangenome 6

The ability of members of the *Pseudomonas* genus to contaminate refrigerated food products has been attributed to 1) the capacity of some strains to grow at low temperatures, and 2) bacterial proteolytic and lipolytic systems that facilitate the breakdown of proteins and fats forming byproducts that are useful for growth and for the production of volatile compounds 5. Food products at the end of their shelf life can serve as excellent substrates for bacterial production of secondary metabolites if the food is rich in free amino acids including phenylalanine (needed for pyomelanin production), glutamine (a precursor to the blue pigments pyocyanin and indigoidine) and aromatic amino acids such as tryptophan which can be used in the production of indole, the violet pigment violacein, and other biosynthetically related pigments 5. Many bacteria can produce indole from tryptophan using the enzyme tryptophanase and can then use oxygenases to oxidize indole and form indigo 7. Additionally, pigment production can vary depending on the medium. For example, *Pseudomonas azotoformans* has been found to form a grey color in pasteurized milk following incubation for long periods at refrigeration temperatures and can generate black pigmentation on Dichloran Rose Bengal agar 8.

**Figure 1:**
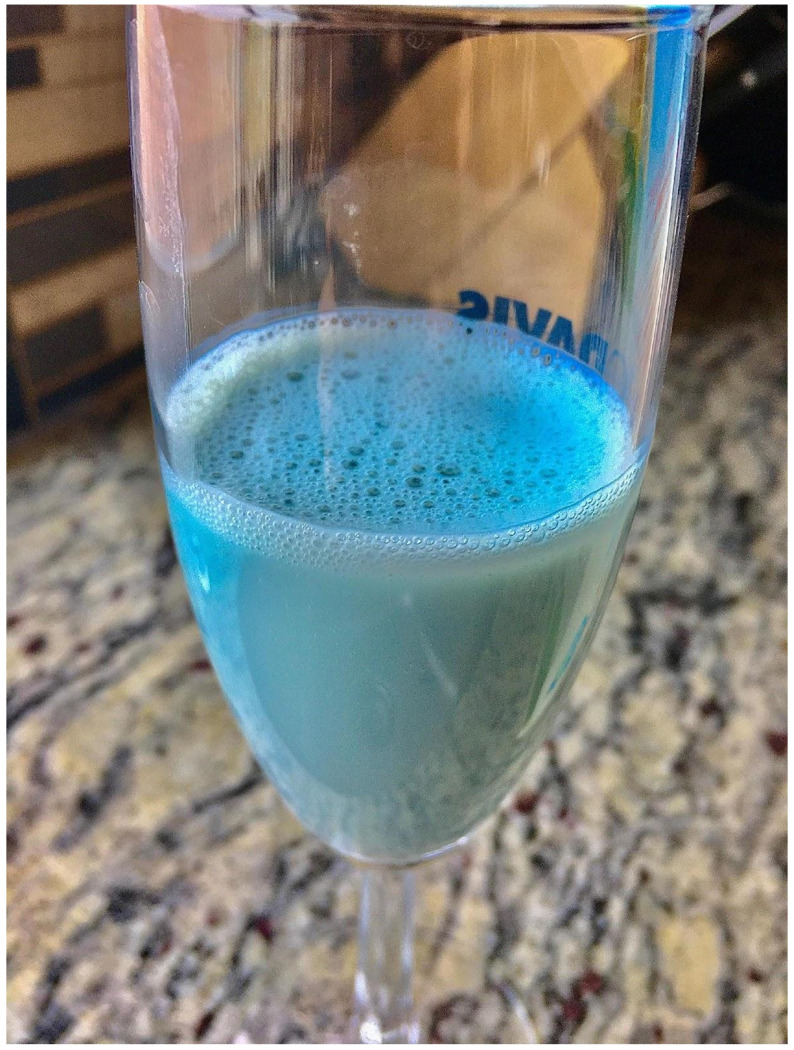
The blue coloration of soymilk was discovered after being poured from an open container into a glass.

**Figure 2:**
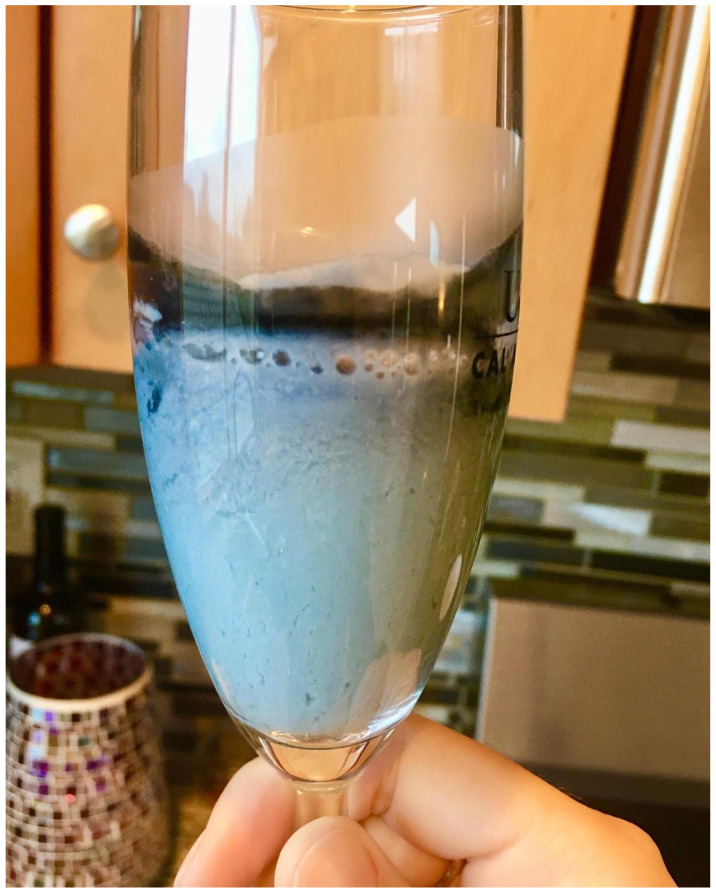
The same blue colored soymilk as shown in Figure 1 after having been left for 2 days at room temperature on a countertop.

**Figure 3:**
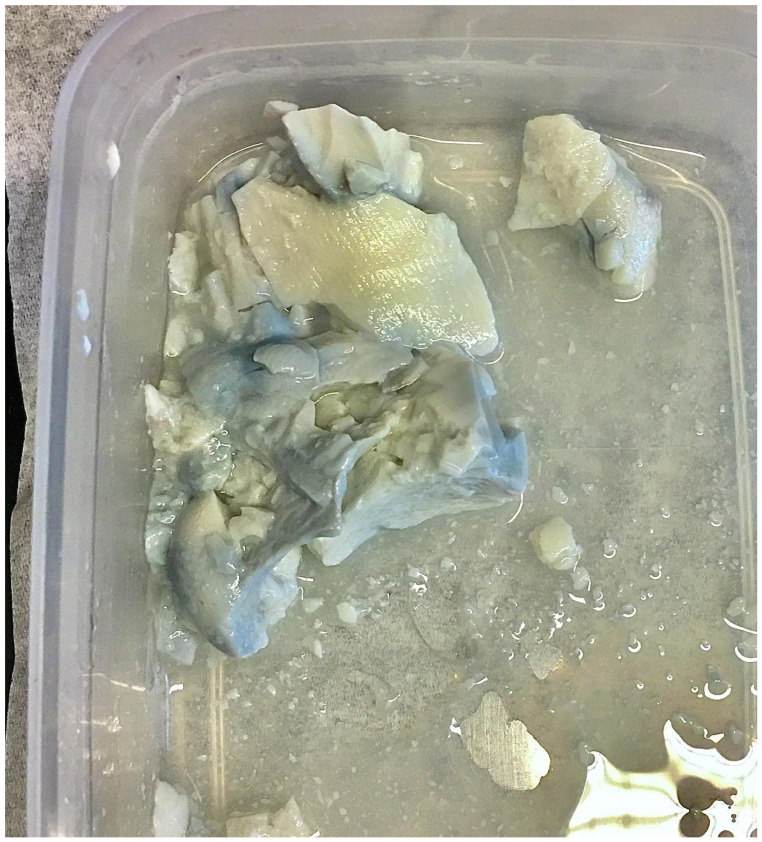
Blue silken tofu was discovered after the package was opened and the tofu was partially used.

We observed a brilliant blue discoloration of partially used soy products left in a refrigerator (Figures 1-3) and then cultured microorganisms from the products in order to analyze the contaminants genetically. Although contamination by *Pseudomonas* strains is relatively common in dairy and meat, blue discoloration of soy products has not yet been reported. We used a combination of culturing under experimental conditions and nucleotide sequencing (16S rRNA and whole genome shotgun) to isolate and genetically characterize four bacterial strains potentially responsible for the blue discoloration of soy products. Specifically, we report the isolation and DNA sequence analysis of two novel *Pseudomonas* strains that are able to cause blue discoloration in soy food products.

## Methods

### Culturing and isolation

After a blue appearance was observed in partially used, expired soy products (milk and tofu) that were stored in a home refrigerator, the defective soy products were brought into the laboratory for investigation into possible microbial contamination. A 100 uL sample of blue soymilk was pipetted onto each of two agar plates (LB and R2A) and spread over the surface using a sterile loop. Samples from the blue/grey colored portion of tofu were collected using a sterile loop and used to inoculate two additional LB and R2A agar plates. All plates were incubated at room temperature (~18-20 °C) for 48 hours. Following observation, successive subcultures were prepared until four pure cultures were established. Samples from isolated colonies were then transferred to Tryptic Soy Agar (TSA) plates for additional observation and cultivation. The four distinct isolates obtained were designated UCD_MED2, UCD_MED3, UCD_MED5 and UCD_MED7. Aliquots of these isolates were frozen in 25% glycerol in cryovials for long-term storage, and were used for additional investigations.

Isolates UCD_MED3 and UCD_MED7 were subcultured on Petri plates and the surfaces of agar deep tubes containing Mueller Hinton agar (MHA), a medium shown to promote pyomelanin production by some bacteria 9,10. Following incubation at room temperature for 48 hours MHA plates and agar deep tubes were observed for production of brown pigment, and also exposed to ultraviolet irradiation to detect fluorescence.

### Testing the ability of isolates to cause blue discoloration in soymilk

Fresh soymilk of the same brand as the soymilk with the observed blue discoloration was purchased, opened, and a 200 mL aliquot was poured into a sterile 500 mL bottle under aseptic conditions. The bottle was loosely capped and the soymilk was boiled for ten minutes. After cooling, 3 mL aliquots of the heat-treated milk were aseptically transferred into eight, sterile 10 mL glass culture tubes. Single colonies from each of the two morphologically distinct soymilk isolates (UCD_MED5 and UCD_MED7) were aseptically transferred into soymilk tubes. A mixed culture of these same isolates was also prepared. Uninoculated, heat-treated soymilk was used as a negative control. These tubes were placed on an orbital shaker (220 rpm) and incubated at room temperature for 48 hours. Following this, tubes were transferred to a 4 °C refrigerator and incubated for an additional four days without shaking.

### DNA extraction and PCR

Template DNA for PCR amplification of 16S rRNA genes was obtained from 24-48 hour plate cultures. A 2 mm sample from an isolated colony of each culture type was boiled for 10 minutes in 500 uL of 10 mM Tris buffer pH 8.5. The PCR reaction mixture included 5 uL of template DNA, 5 uL of primer mix (10 uM dilution of each primer), 25 uL of *Taq* Master mix (Qiagen) and 15 uL of sterile water (Qiagen). The PCR was conducted as recommended (Northup et al 2003) with modifications specified by Qiagen technical support personnel. Thermal cycler conditions were as follows: 4 minutes denaturation at 94 °C, followed by 35 cycles of 45 seconds at 55 °C, 2 minutes at 72 °C, and 30 seconds at 94 °C, with a final 45 seconds at 55 °C, and 20 minutes at 72 °C. The PCR primers used were Bacteria 8-forward (5'-AGAGTTTGATCCTGGCTCAG-3') and Enteric 1530-reverse (5'-AGGAGGTGATCCARCCGCA-3') (Hugo et al., 2003). Following PCR amplification, each 50 uL sample was visualized through agarose gel electrophoresis, the DNA cut from the gels manually and purified using QIAquick gel purification kits (Qiagen). Purified DNA samples (16 uL each) were delivered to the University of California, Davis. Sanger sequencing, using 1-2 uL per reaction, was performed on a 3730 Capillary Electrophoresis Genetic Analyzer and ABI BigDye Terminator v3.1 chemistry at the UC Davis DNA Sequencing Facility. Sequencing primers included those indicated above plus Internal 533-forward (5'-CCAGCACGCCGCGGTAA-3') and 907-reverse (5'-CCGTCAATTCMTTTRAGTTT-3').

For extraction of genomic DNA used for whole genome sequencing, single colonies from plate cultures of UCD_MED3 and UCD_MED7 were transferred into 5 mL aliquots of tryptic soy broth and incubated for 24 hours on an orbital shaker (220 rpm) at room temperature. DNA was extracted from these cultures using a Qiagen DNeasy Blood and Tissue kit following manufacturer instructions.

### 16S rRNA gene sequencing

Raw sequence data (.ab1 files) were opened with 4Peaks (RRID:SCR_000015) and traces were observed to determine quality and length (typically 800-900 nucleotides). Reads generated with reverse primers were “flipped” to obtain reverse complementary sequences and overlapping regions were compared visually. Nucleotide sequences were copied to a Word file for additional comparison and editing. The four sequences were concatenated and overlapping sections deleted. The regions of the sequence corresponding to the PCR primers were identified and removed.

### Taxonomic and phylogenetic analysis

Initial genus level taxonomic assignment was determined for each of the four isolates based on 16S rRNA gene sequence comparisons done using the NCBI BLAST web server to search the reference RNA sequences (refseq_rna) 11. The 16S rRNA genes of isolates UCD_MED2 and UCD_MED5 were most similar to those from organisms in the genus *Serratia*. The 16S rRNA genes from UCD_MED3 and UCD_MED7 were most similar to those from organisms in the genus *Pseudomonas*. We then downloaded the 16S rRNA gene sequences for type strains in the genus *Serratia* that are available via the Ribosomal Database Project (RDP) 12. We used the 16S rRNA gene sequence of UCD_MED3 as an outgroup. A multiple sequence alignment was then made using all of these sequences and also those of UCD_MED2 and UCD_MED5 applying the NG Phylogeny (ngphylogeny.fr) GUI with MAFFT version 7 and default options 13,14; BMGE 15 was used to extract relevant information from the MSA, and the output tree was built using FastMe version 2.0 16. Finally, the tree was viewed using iTOL 17.

### Genome sequencing and assembly

DNA extracted from isolates UCD_MED3 and UCD_MED7 was quantified using a Qubit 4 Fluorometer and the volumes and concentrations specified were submitted to MicrobesNG, Birmingham, UK for sequencing and assembly. The processes used by MicrobesNG for this are as follows: (a) whole genome shotgun sequences were generated on an Illumina NovaSeq 6000 with 2 x 250 bp paired-end reads (b) the closest available reference genomes were determined using Kraken 18, (c) reads were trimmed using Trimmomatic 19, (d) quality was assessed using MicrobesNG in house scripts along with Samtools, Bedtools and BWA-mem (e) de novo assemblies were generated using SPAdes version 3.7 20.

The collection of contigs greater than 199 bp generated for each isolate were submitted to the National Center for Biotechnology Information (NCBI) Prokaryotic Genome Annotation Pipeline (software revision 3.3) 21. The genomes of UCD_MED3 and UCD_MED7 (all contigs greater than 199 bp) along with the NCBI annotation were submitted to GenBank and given accession IDs GCA_019097565.1 and GCA_019097535.1, respectively.

### Whole genome phylogeny

To compare and taxonomically place strains UCD_MED3 and UCD_MED7, we gathered additional genomes from (1) *Pseudomonas* isolates described in literature as being blue pigmented as well as related isolates from the same studies that were reported not to produce a blue pigment under the same growth conditions; *Pseudomonas* strains Ps_22, Ps_77, FSL_E2-0548, FSL_R10-2514, FSL_E2-8864, FSL_W5-0203, NRRL_B-251, NRRL_B-252, vs Ps_20, Ps_40, FSL_R5-0199, FSL_W7-0098, and FSL_W5-0299, (2) taxa identified based on NCBI and 16S rRNA gene sequence similarities as being potentially closely related to UCD_MED3 and UCD_MED7; *Pseudomonas* strains J380, and myb193, (3) taxa identified based on average nucleotide identity (ANI) similarity; *P. carnis* strain SpeckC, and (4) an additional member of the *Pseudomonas* group that was of interest; *P. paracarnis* strain V5-DAB-2-5. This set of genomes, including UCD_MED3 and UCD_MED7 was placed on a whole genome tree using GTDB-tk v1.5.0, release202 22. The default output tree [pplacer v1.1.alpha19-0-g807f6f3] was pruned to only include 35 nodes away from UCD_MED3 and UCD_MED7 using R 3.2.3 23.

### Quantifying number of homologs of proteins in the tryptophan and pyomelanin synthesis pathways

#### Tryptophan

The genomes from UCD_MED3 and UCD_MED7 plus twenty additional genomes comprising the potential blue branch of the *Pseudomonas* phylogenetic tree were selected to screen for the number of homologs encoding proteins in the tryptophan synthesis pathway. These genomes were identified and downloaded as GBFF files that included their annotation. The predicted proteins for these genomes were extracted using gbseqextractor. These proteins and the predicted proteins for UCD_MED3 and UCD_MED7 were combined into a single file and converted into a BLAST searchable database using makeblastdb. This database was searched with BLASTp for homologs of proteins previously identified as having multiple homologs in the genomes of blue pigment-producing *Pseudomonas* species 24. The specific query sequences used were proteins from a *Pseudomonas* strain known to produce blue pigment (Ps_77) as follows: tryptophan synthase alpha chain (TrpA_PFLuk1_00666), tryptophan synthase subunit beta (TrpB_PFLuk1_00667), indole-3-glycerol phosphate synthase (TrpC_PFLuk1_00676), anthranilate phosphoribosyltransferase (TrpD_PFLuk1_00664), and N- (5′-phosphoribosyl) anthranilate isomerase (TrpF_PFLuk1_0066) 24.

#### Homogentisate

We searched the genomes of UCD_MED3 and UCD_MED7 for homologs of proteins that are known to be involved in homogentisic acid-based synthesis that can lead to the production of pyomelanin. For this searching we used proteins from *Aeromonas media* strain WS which have been shown to have such functional roles: PhhA_B224_2628 phenylalanine-4-hydroxylase, PhhB_B224_2627 pterin-4-alpha-carbinolamine dehydratase, AspC_B224_3173 aromatic-amino-acid aminotransferase, HppD_B224_2891 4-hydroxyphenylpyruvate dioxygenase, and TyrB_B224_5900 aromatic-amino-acid aminotransferase 25. These were searched with BLASTp against a mini database of proteins encoded by UCD_MED3 and UCD_MED7 that were combined into a single searchable database using makeblastdb. Matches with E-value scores of better than 1×E^-10^ were considered likely homologs and were examined.

## Results

### Isolation of strains from discolored soymilk and tofu

**Figure 4:**
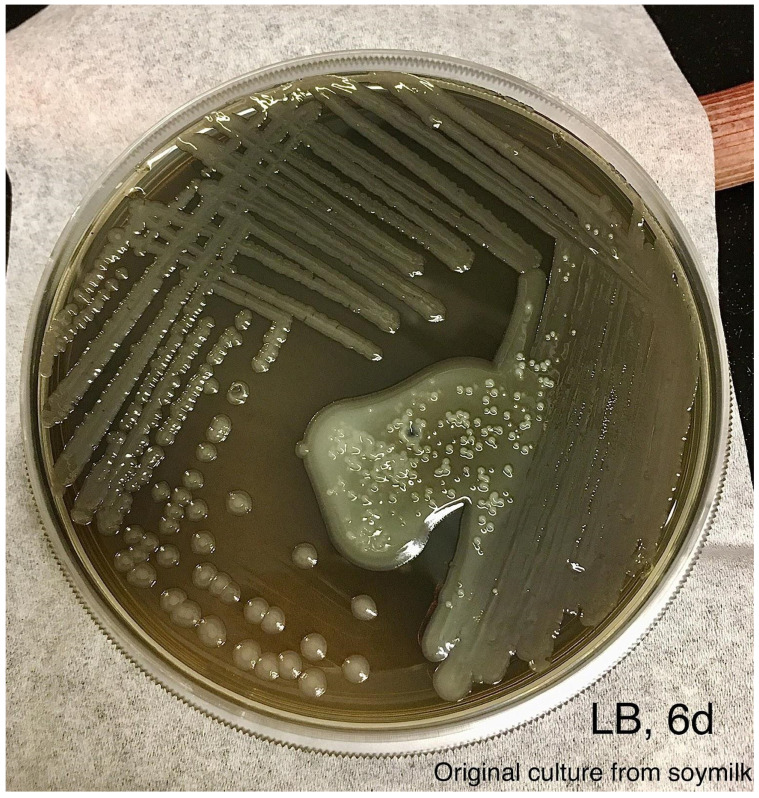
An aliquot of the blue colored soymilk was pipetted onto LB agar and streaked for single colonies. Photo was taken after incubation at room temperature for 6 days.

**Figure 5:**
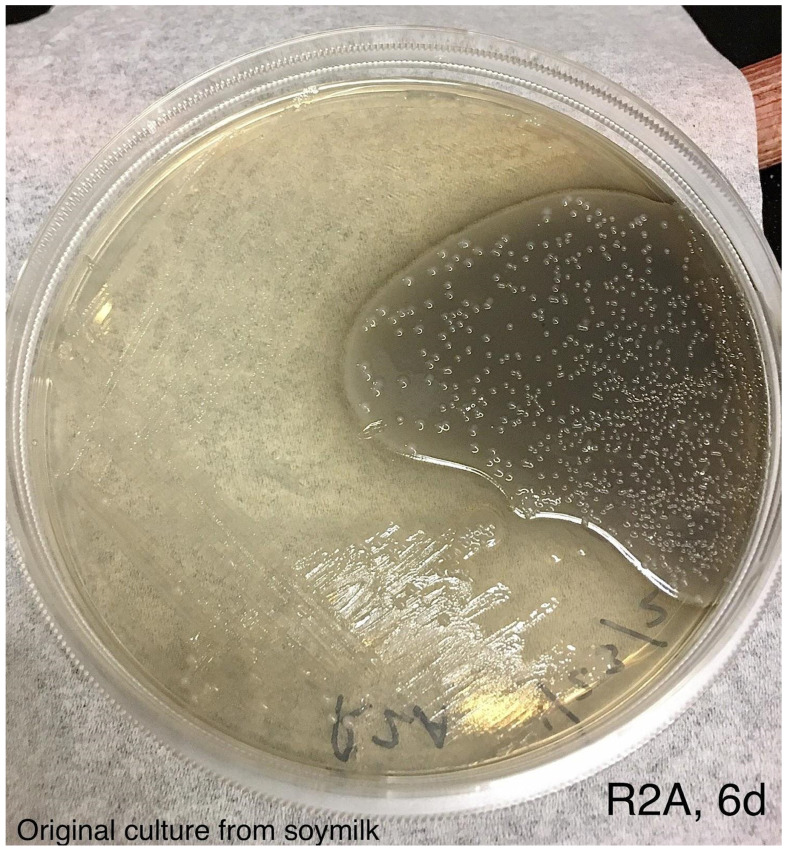
An aliquot of the blue colored soymilk was pipetted onto R2A agar and streaked for single colonies. Photo was taken after incubation at room temperature for 6 days.

**Figure 6:**
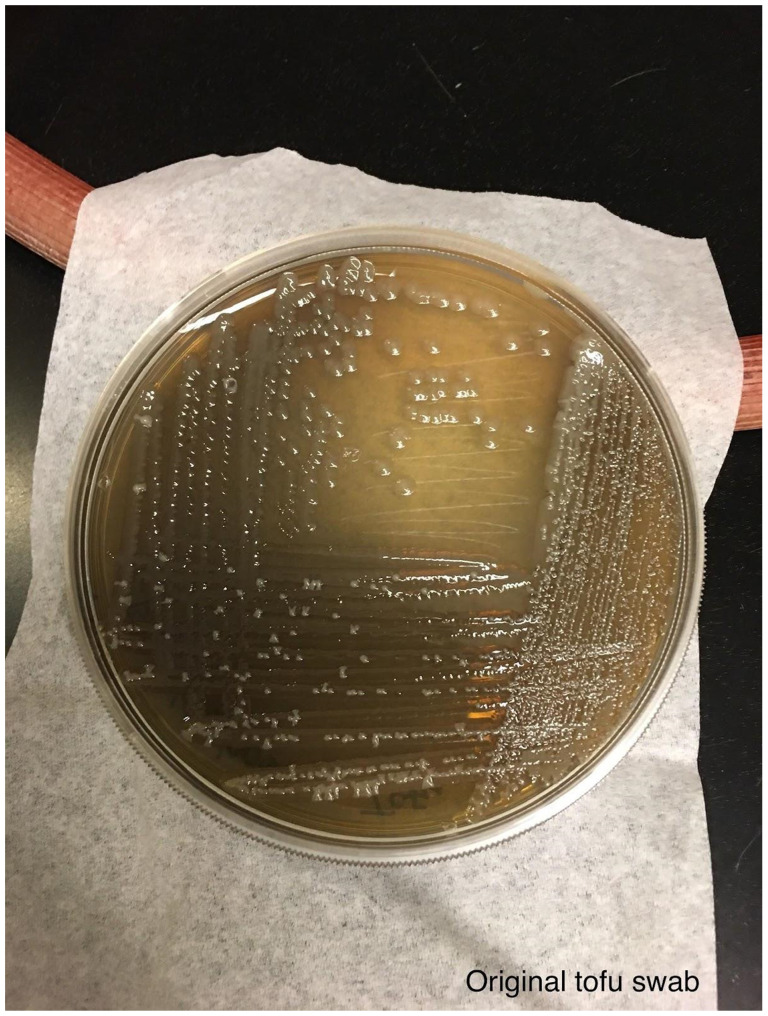
An aliquot of the blue colored tofu was spread using a sterile loop onto LB agar and streaked for single colonies. Photo was taken after incubation at room temperature for 6 days.

**Figure 7:**
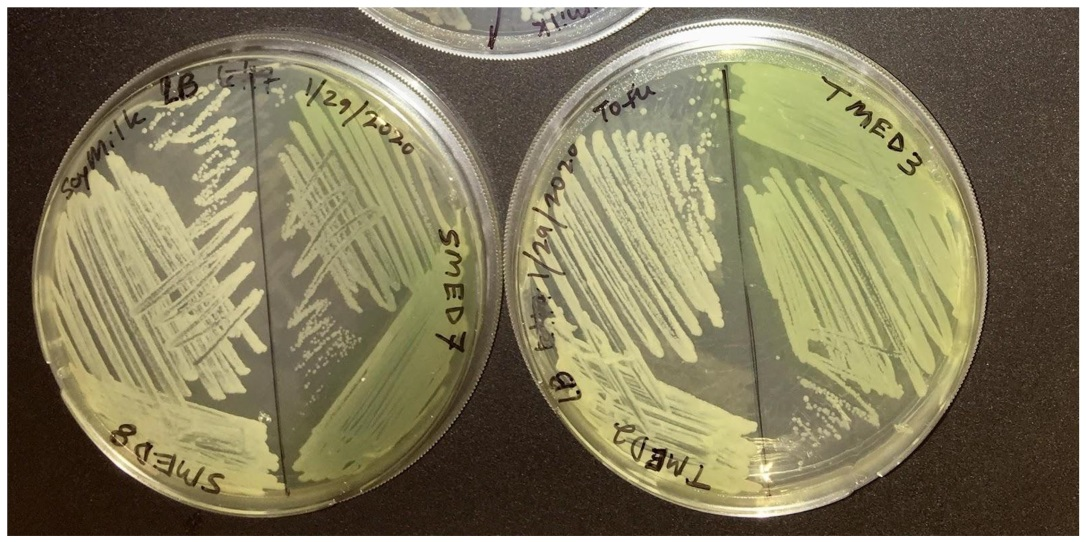
Restreak for isolation on LB agar (UCD_MED5 is not shown). Strains UCD_MED2, UCD_MED3, UCD_MED7, and UCD_MED8 (replicate strain UCD_MED8 was omitted from further analysis). Cultures were incubated at room temperature for 48 hours.

**Figure 8:**
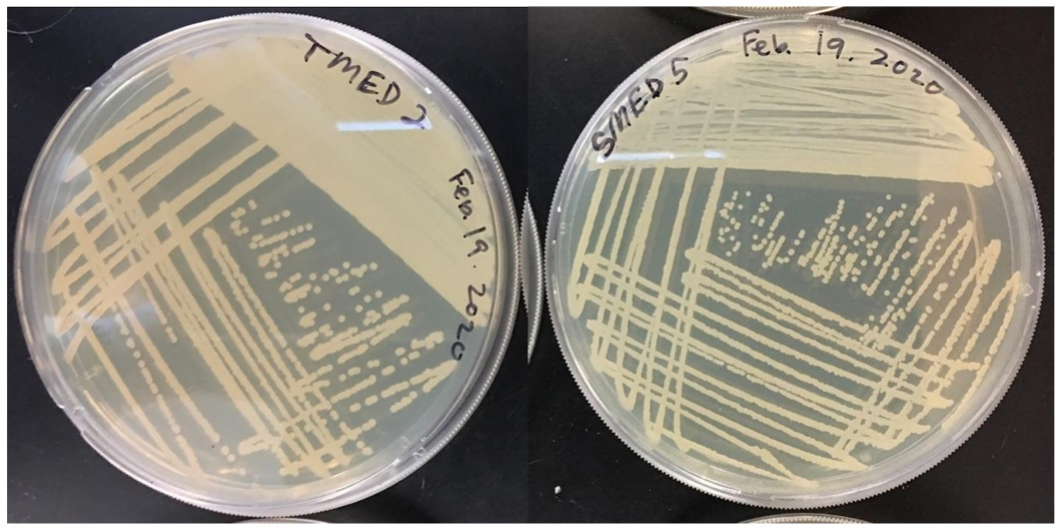
Restreak for isolation on R2A medium. Left; UCD_MED2, Right; UCD_MED5. Cultures were incubated at room temperature for 48 hours.

**Figure 9:**
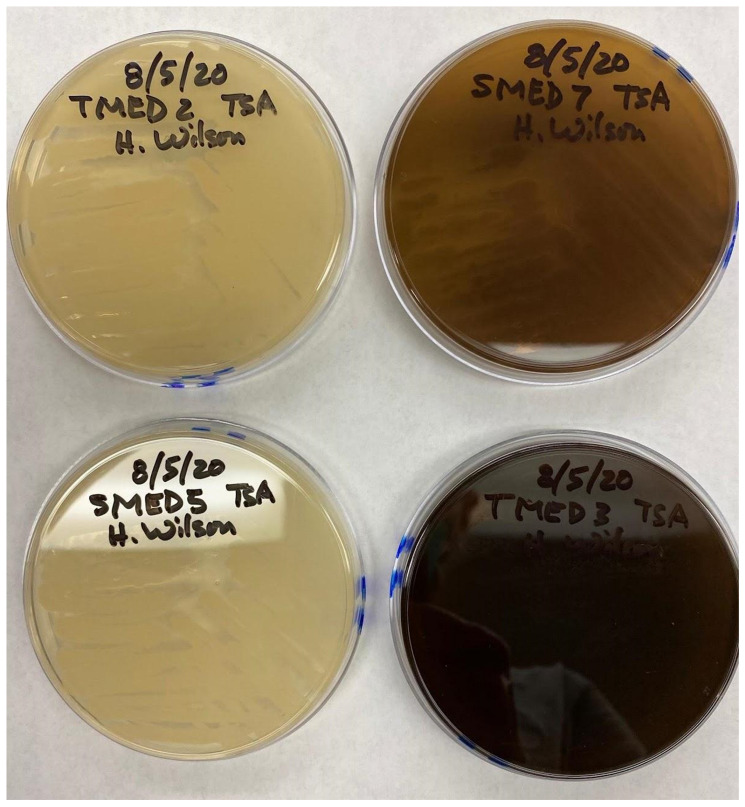
Growth on TSA medium. Clockwise from top left; UCD_MED2, UCD_MED7, UCD_MED5, and UCD_MED3. Cultures were incubated at room temperature for 48 hours.

When samples from blue-colored soy products were initially cultured on solid media, two distinct growth forms were evident: scattered, small, opaque-white colonies, and a semi-opaque lawn (Figures 4-6). Regions with different appearances formed distinctly different-looking isolated colonies when subcultured on new media (LB, R2A and TSA). These were either semi-opaque and greenish in color or opaque and whitish-yellow in color on R2A and LB plates, and semi-opaque and brown or opaque and creamy-white on TSA (Figures 7-9). Two distinct types of colonies were selected from each of the initial plate cultures, and these were transferred to new media for isolation and characterization. Two of the cultures eventually produced a greenish or brownish pigment that diffused into and changed the color of the agar; the other two colonies were creamy-white in color and did not produce a visible diffusible pigment. In total we selected four isolates (2 from each soy substrate) for further characterization (Table 1).

### Culturing for other pigment production

**Figure 10:**
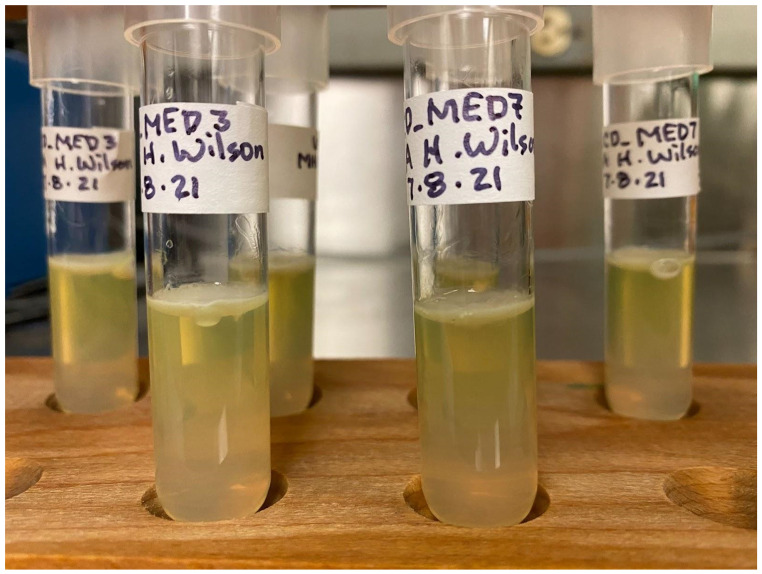
Growth of UCD_MED3 and UCD_MED7 on MHA deeps. Bacterial growth occurs largely at the surface of the agar and the yellow-green pigment diffuses deep into the agar.

**Figure 11:**
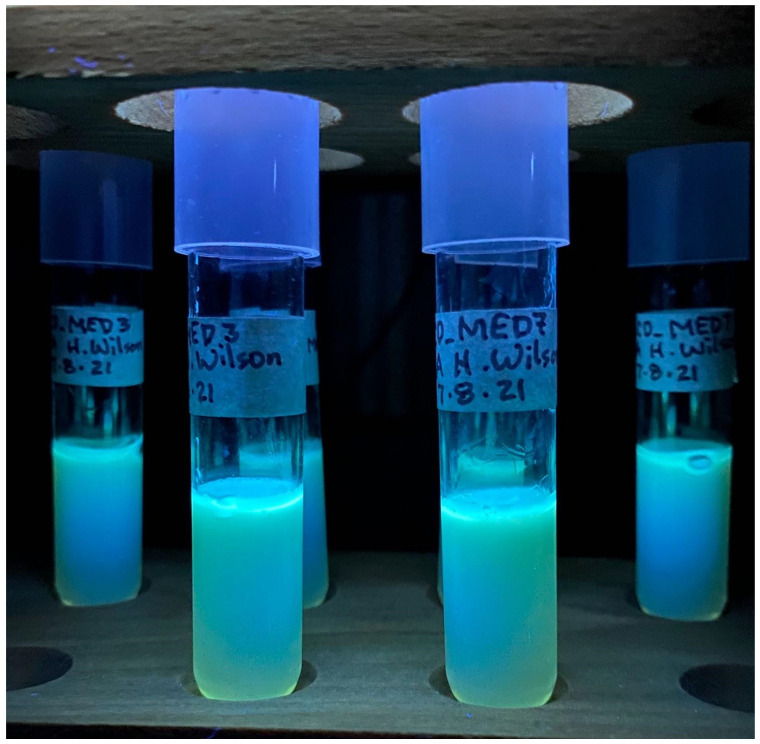
Exposure of UCD_MED3 and UCD_MED7 to ultra-violet irradiation causes the bacteria and the pigment to fluoresce.

After 48 hours of growth in MHA deeps and on plates, UCD_MED3 and UCD_MED7 strains formed pale, yellow-green colonies. In addition, the originally colorless agar also changed to pale, yellow-green throughout. The bacteria fluoresced when exposed to ultraviolet irradiation (Figures 10 and 11), and after 72 hours of growth, a small amount of brown pigment developed, however the intensity of brown coloration was lower than when the bacteria were cultured on TSA medium.

### Tests for blue pigment production

**Figure 12:**
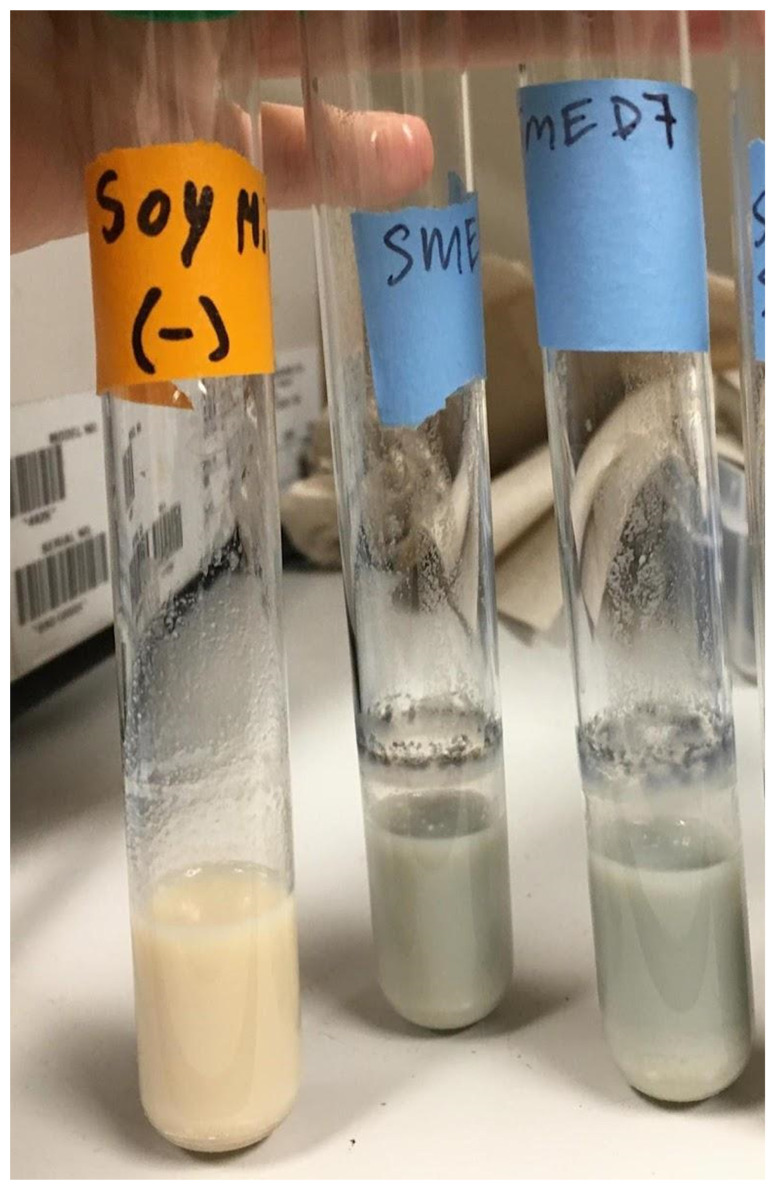
Soymilk cultures after 48 hours incubation at room temperature, shaking. Left; uninoculated soymilk (single test tube). Right (two test tubes); soymilk inoculated with isolate UCD_MED7.

**Figure 13:**
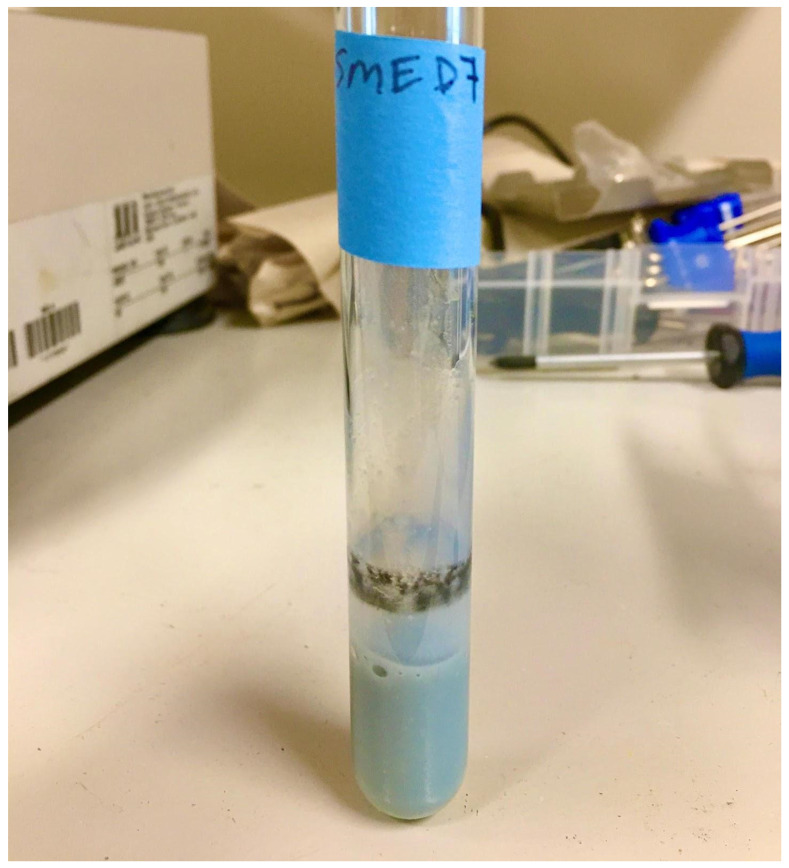
Strain UCD_MED7 after 4 days incubation in sterile soymilk at 4 °C.

**Figure 14:**
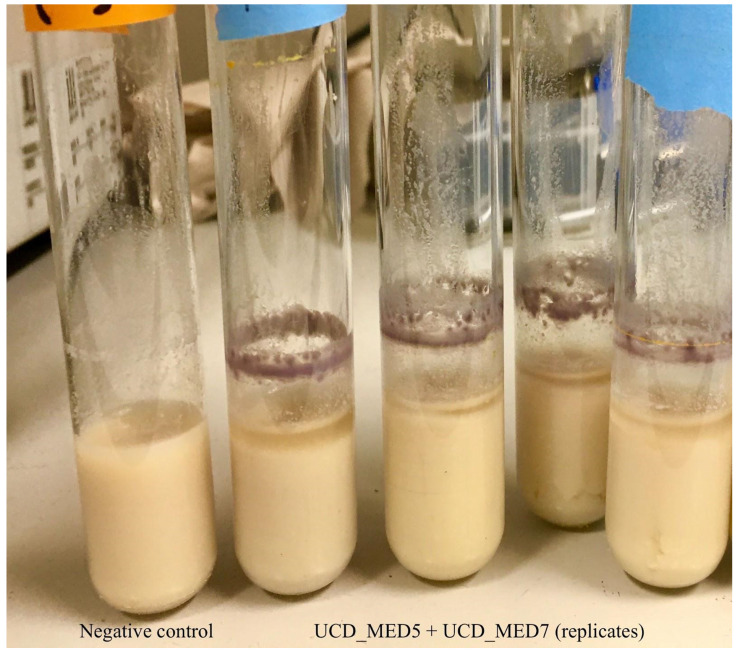
Left tube; negative control (sterile soymilk only). Right tubes; coculture of strains UCD_MED5 + UCD_MED7 in sterile soymilk after 4 days incubation at 4 °C.

Heat-treated soymilk inoculated with isolate UCD_MED5 showed signs of growth, but no color change after 48 hours of incubation at room temperature nor after refrigeration at 4 °C for an additional four days. Soymilk inoculated with isolate UCD_MED7 developed a grey tint and had a black biofilm forming at the liquid-air interface after 24 hours of incubation at room temperature (Figure 12). When moved to the 4 °C refrigerator and incubated for an additional four days the soymilk turned a brilliant blue color (Figure 13). The coculture of isolates UCD_MED5 and UCD_MED7 formed a visible, milky biofilm at the liquid-air interface after 24 hours incubation at room temperature and no color change was observed. After four additional days of incubation at 4 °C the biofilm turned light purple/lavender in color, however the color of the liquid soymilk remained unchanged (Figure 14). No growth was observed in the uninoculated soymilk used as a negative control (Table 2).

### Taxonomic analysis of isolates from 16S rRNA gene sequence data

The 16S rRNA genes of isolates UCD_MED3 and UCD_MED7 showed a top match using BLASTn to the 16S rRNA genes in a complete genome from *Pseudomonas sp.* strain J380 (RefSeq accession number GCF_009827115.1). The 16S rRNA genes of isolates UCD_MED2 and UCD_MED5 showed a top match using BLASTn to multiple 16S rRNA genes found in two complete genomes: *Serratia liquefaciens* strain FG3 (RefSeq accession number GCF_006970665.1) and strain S1 (RefSeq accession number GCF_008364325.2), respectively (Table 3).

**Figure 15:**
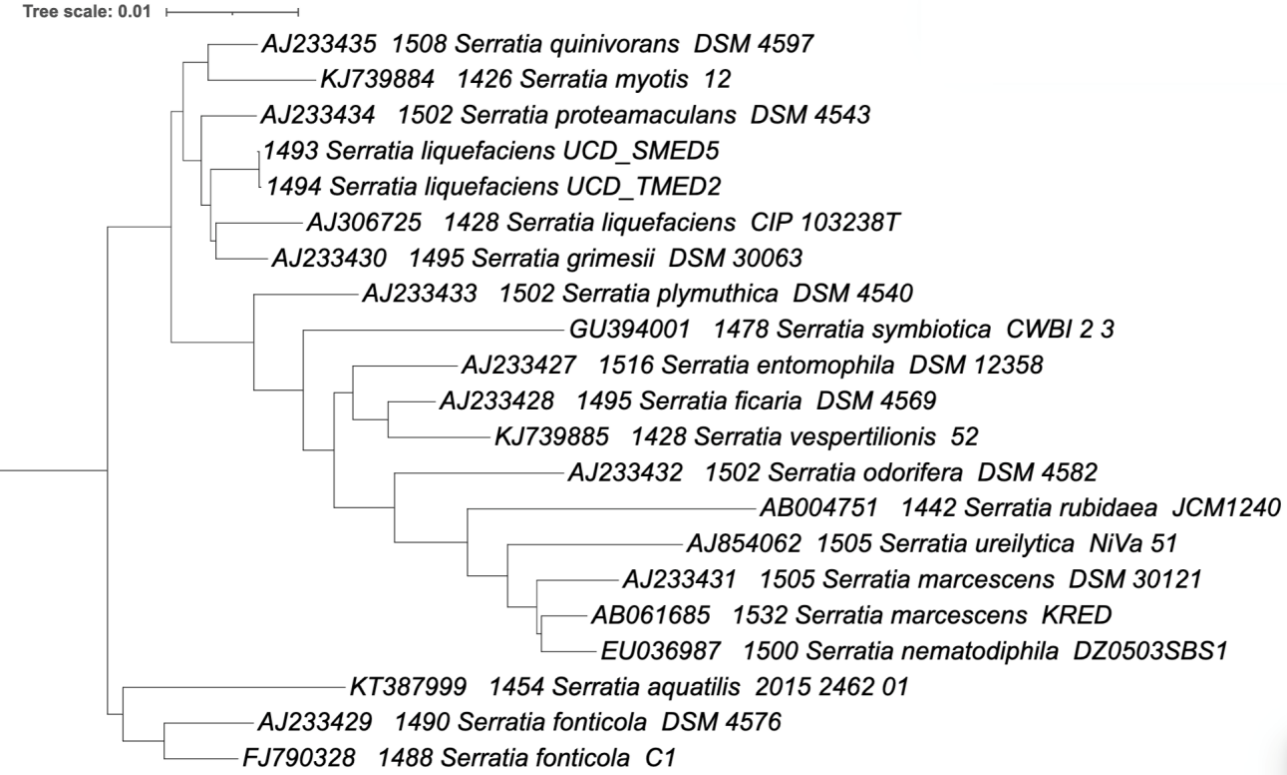
Phylogenetic tree based on 16S rRNA gene for type strains in the *Serratia* genus with UCD_MED2 and UCD_MED5 gene sequences included. *Pseudomonas* strain UCD_MED3 was used as an outgroup to root the tree (not shown). Branch length units are based on nucleotide substitutions per site. Leaf label organization; RDP ID, length (bp), specific epithet, strain ID.

To better characterize the taxonomy of isolates UCD_MED2 and UCD_MED5, we carried out a phylogenetic analysis of 16S rRNA gene sequences using *Serratia* species type strains available in RDP. Both strains UCD_MED2 and UCD_MED5 are sister taxa to *S. liquefaciens* strain CIP 103238 and *S. grimesii* strain DSM 30063 (Figure 15).

### Genome sequencing, assembly, and quality

The Illumina whole genome shotgun sequencing for UCD_MED3 generated 1,261,370 reads and for UCD_MED7 generated 1,750,514 reads. The reads were assembled into 88 contigs for UCD_MED3 and 82 contigs for UCD_MED7 (Table 4). The genome quality evaluation initiated through CheckM 26 generated 308 clade-specific marker gene sets for each genome assembly. The genome of UCD_MED3 showed 100% completeness (all marker genes were present) and 0.89% contamination (measured based on % of extra homologs of some of these genes), while that of UCD_MED7 showed 100% completeness and 1.27% contamination.

### Comparative and phylogenetic analysis of UCD_MED3 and UCD_MED7 genomes

**Figure 16:**
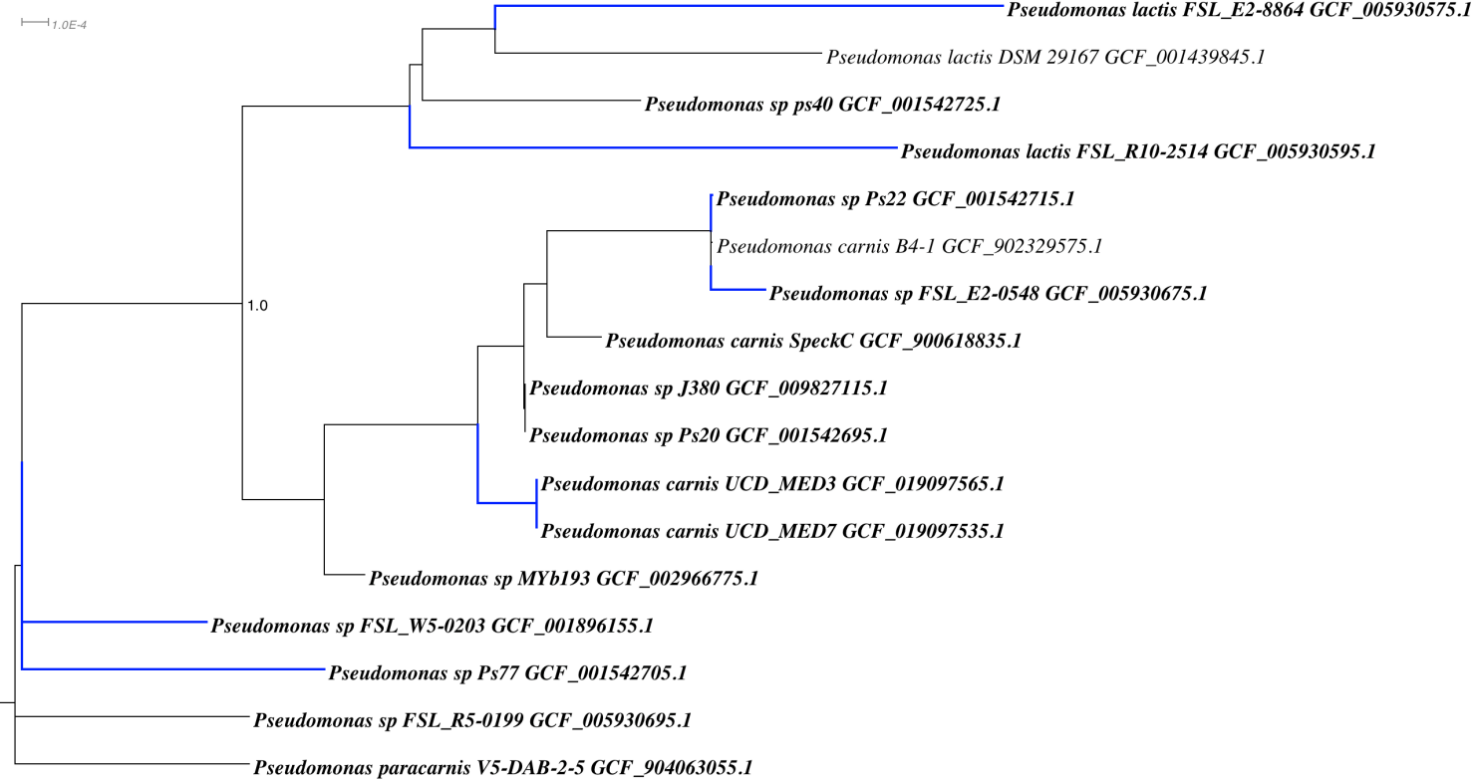
GTDB-tk phylogenetic tree based on genomes of Pseudomonas strains, including UCD_MED3 and UCD_MED7. Strains in bold print were added to the GTDB-tk tree manually. Stains with blue branches have been shown to produce blue pigment.

Whole genome analysis was used to establish a phylogenetic context for placing these strains in the “blue clade” and also revealed patterns of multiple homologs associated with the tryptophan biosynthesis pathways similar to those reported for other blue discoloration causing members of the *Pseudomonas* group. A whole genome based Genome Taxonomy DataBase (GTDB) phylogenetic tree including UCD_MED3 and UCD_MED7 and additional related bacteria is shown in Figure 16. This tree indicates the two new isolates are most closely related to existing *Pseudomonas carnis* strains and that they are sister taxa to the *Pseudomonas lactis* strain DSM 29167 (type strain) and other strains described as *P. lactis*. UCD_MED3 and UCD_MED7 shared an ANI value of 98.46% with *P. carnis* strain SpeckC (GenBank accession number GCA_900618835.1), while the ANI value between UCD_MED3 and UCD_MED7 isolates and the *P. lactis* type strain approaches 95% at 94.91%.

### Homologs of genes in tryptophan synthesis pathway

The presence of multiple homologs of a set of genes normally involved in tryptophan synthesis has been shown to be a common feature of blue pigment producing *Pseudomonas* strains 24. We tabulated the number of homologs for a set of five of these key genes in 22 genomes. Searches were conducted at the protein level because the identification of homologs works better with protein searchers than with gene searches. A homolog was considered present if there was a hit with an E-value better than 1×E^-10^. Ten isolates confirmed to produce blue pigment contain at least two homologs of each gene 24,27. Two isolates contain two homologs of each *trp* gene but have not been reported to produce blue pigment 28. Five isolates that have not been reported to produce a blue pigment contain only one homolog of each gene 28,29. In addition, five isolates for which blue pigment production was tested but not observed contain only one gene 24,27 (Table 5).

### Homogentisate synthesis gene homologs

Homogentisate based pyomelanin production by *A. media* has been shown to require *phhA, phhB, aspC, hppD,* and* tyrB* genes which constitute a linear pathway for converting phenylalanine to HGA 25. We searched UCD_MED3 and UCD_MED7 for protein sequences found to be required for pyomelanin production in *A. media* genomes that are available in NCBI. Both UCD_MED3 and UCD_MED7 contained at least one copy of each of these proteins (Table 6).

### Data availability

The draft genome sequences of the new isolates *Pseudomonas* strains UCD_MED3 and UCD_MED7 (Genome assembly accession numbers: GCA_019097565.1 and GCA_019097535.1). The whole genome sequence accession numbers for UCD_MED3 and UCD_MED7 are JAGTYD000000000 and JAGTYE000000000, respectively. The 16S rRNA gene sequences for the* Serratia liquefaciens* UCD_MED2 and UCD_MED5 isolates are listed under GenBank nucleotide sequence ID accession numbers: UCD_MED2; MZ027635.1, and UCD_MED5; MZ027636.1, respectively. This project has been deposited under the accession PRJNA721694. The version described in this paper is version PRJNA721694. All strains are available from the corresponding author upon request. All bacterial isolates are maintained in 25% glycerol frozen stocks and are available for future studies through the UC Davis Laboratory collection of Jonathan A. Eisen.

## Discussion

Our work as described here focused on isolating and genetically characterizing organisms likely associated with the distinctly blue discoloration observed in spoiled soy and tofu products. We isolated and genetically characterized (at various levels of detail) four bacterial strains we designated as UCD_MED2, UCD_MED3, UCD_MED5, and UCD_MED7. When the *Pseudomonas* isolates were cultured on various media, colonies developed different colors. When incubated at room temperature for 48 hours on LB medium the bacteria formed bright yellow-green colonies. When incubated in the same conditions on TSA, a brown pigment developed that diffused into the agar turning the agar dark brown in addition to the colonies. The yellow-green color resembled a type of fluorescein or pyoverdine and the brown color resembled pyomelanin. Depending on the media, These *Pseudomonas* strains isolated from refrigerated, expired soy foods produce a wide range of colors that could have interesting uses in the food industry.

### Serratia

Two of the isolates, UCD_MED2 and UCD_MED5, showed similar cultural characteristics although initially taken from different substrates (Figures 8 and 9). Of these, UCD_MED5 (isolated from soymilk) did not appear to cause blue discoloration under experimental conditions. A mixed culture of UCD_MED5 and UCD_MED7 incubated for four days at 4 °C formed a light purple or lavender biofilm (Figure 13). This chromatic phenomenon, seemingly induced by an interaction between the two organism types, would be an interesting ecological study to explore in the future. Phylogenetic analysis of UCD_MED2 and UCD_MED5 revealed both to be members of the genus *Serratia*. A phylogenetic tree indicating their position within type strains of this genus is shown in Figure 15. Based on organization of this tree and 16S rRNA closest sequence similarity, we have assigned isolates UCD_MED2 and UCD_MED5 to the species *Serratia liquefaciens*. Members of the genus *Serratia* have been found in diverse environments including refrigerated food products, soil, air, water, and on plant surfaces 30-32.

### Pseudomonas

The isolates designated as UCD_MED3 and UCD_MED7 formed colonies with nearly identical features. Only strain UCD_MED7 (isolated from soymilk) was used for experimentation. Our studies showed that while UCD_MED7 can proliferate in soymilk at room temperature, it produces intense, blue pigmentation only when incubated for a longer time period at refrigeration temperatures (Figure 13). Since the color formed during experimentation appeared identical to the original color of the defective soymilk, we conclude that the presence of UCD_MED7 is the probable cause for the blue discoloration in the soy products. We cannot rule out the possibility that the presence of other organisms including UCD_MED5 could have contributed to discoloration considering that the coculture of UCD_MED5 and UCD_MED7 formed a purple/lavender biofilm. We note that UCD_MED7 produced a black biofilm at the surface of the liquid soymilk and that both UCD_MED3 and UCD_MED7 produced a dark, water-soluble pigment when cultured on TSA. These characteristics have been reported for the related species *P. carnis* in different types of media 28.

We sequenced and analyzed the genomes of UCD_MED3 and UCD_MED7 to gain insight into the classification, phylogeny and function of these unique strains. Genome based phylogenetic analysis and examination of ANI indicates that UCD_MED3 and UCD_MED7 are members of the genus *Pseudomonas*. We refer to these two new strains as *Pseudomonas carnis* strain UCD_MED3 and *Pseudomonas carnis* strain UCD_MED7 due to their phylogenetic placement with the *P. carnis* species type strain B4-1. They are in a sister group to *Pseudomonas lactis* and *Pseudomonas paracarnis*. Additionally, *P. lactis* and *P. fluorescens* are among those species previously reported as present in blue-colored dairy products such as milks, cheeses and queso fresco 27,33. *Pseudomonas carnis* UCD_MED3 and *Pseudomonas carnis* UCD_MED7 lie within a highly blue pigmented clade, however some other species designations in this clade remain debatable, and strains that produce a blue pigment are found outside of this main clade.

### *trpABCDF* genes

Prior studies of blue discoloration in refrigerated foods caused by *Pseudomonas* species has led to the discovery that the blue pigment producing bacteria have multiple, divergent homologs of genes in the tryptophan biosynthesis pathway (specifically *trpABCDF*) and non-pigment-producing strains contain only one homolog of each gene 24. We carried out an analysis of the *P. carnis.* UCD_MED3 and *P. carnis* UCD_MED7 genomes together with a collection of related taxa for the presence and number of homologs of these *trp* genes.

*P. carnis* strains UCD_MED3 and UCD_MED7 both contain at least two homologs of each of the *trpABCDF* genes. We also detected multiple homologs of these genes in confirmed blue pigment-producing *Pseudomonas* strains, as well as two *Pseudomonas carnis* strains that have been reported to produce black and grey diffusible pigments on multiple types of media, including refrigerated pig flesh 24,27,28. These results corroborate previous findings and suggest that our analysis is comparable to others. The genome of a confirmed blue compound producing isolate Ps_22 contains only one homolog of *trpB;* however, it has been suggested that there may be a second homolog that was missed due to low coverage of the genome 24. Two strains of *P. carnis* (SpeckC and B4-1) are reported to produce a black, diffusible pigment on Czapek yeast extract agar, and a grey biofilm layer forms at the liquid-air interface in Standard-I bouillon or Caso bouillon broth 28. There are no reports of these strains producing blue pigment, however there are also no reports we could find of tests for such production. Both strains contain multiple homologs of each *trp* gene, and therefore we predict they are likely capable of blue pigment production given favorable substrate and/or environmental conditions such as refrigeration and exposure to oxygen. Interestingly, while most of the recognized blue pigment producing *Pseudomonas* strains organize within a single clade within the GTDB phylogeny, two blue pigmented strains (NRRL_B-251 and NRRL_B-252) are more closely related to *P. putida* strains, which are notably distant from the other known blue pigmented bacteria.

The exact mechanism by which *Pseudomonas* species, including UCD_MED3 and UCD_MED7 produce blue pigment is not known. We note that tryptophan metabolism is commonly involved in pigment production, especially blue and violet such as indigo and violacein 5. However, the proposed pathway for indole production involves the conversion of tryptophan to indole by tryptophanase 7. Upon inspection of the protein annotation files (.faa files downloaded from NCBI genome assemblies), we found that neither UCD_MED7 nor UCD_MED3 contain a protein annotated as tryptophanase. Therefore, we hypothesize that the bright blue pigment produced by bacteria in our study is not likely to be indigo or an indigo-derivative molecule unless there is an unknown enzyme present that is also capable of converting tryptophan to indole. The identity of the blue compound secreted by *P*.* carnis* UCD_MED7 and UCD_MED3 s and other strains analyzed in this study remains elusive.

### Pigment production

Given the multiple different colors associated with *P. carnis* strains UCD_MED3 and UCD_MED7 when cultured on various substrates, we believe these strains either have the ability to produce multiple, and some possibly novel pigments, or a variety of different pigments share a common biosynthetic pathway and can lead to color variations when organisms are exposed to different nutrients and/or temperatures. If this is true, it demonstrates the need to consider multiple different substrates when growing isolates in pure culture and describing their morphology. Investigating genetic potential is an important aspect of understanding bacterial pigment biosynthesis and the potential functions of compounds formed.

Pyomelanin is a water-soluble, dark brown pigment produced by various bacteria including some *Pseudomonas* species, and synthesis of this compound results from exposure to tyrosine or phenylalanine in the environment that can be taken up, degraded, and utilized to accumulate homogentisic acid (HGA) 10,34,35. Like other melanins, pyomelanin is formed by the enzymatic oxidation and subsequent polymerization of phenolic and/or indolic compounds 25,36. When grown on TSA for just 48 hours, UCD_MED3 and UCD_MED7 produced a dark brown diffusible pigment resembling pyomelanin, and through genomic analysis we found that these strains contain homologs of genes that in other organisms have been shown to be necessary for the production of pyomelanin. Therefore, we conclude that *P*.* carnis* strains UCD_MED3 and UCD_MED7 are likely able to carry out the same biosynthetic process.

*P*.* carnis* UCD_MED3 and UCD_MED7 also produced a pale, yellow-green, diffusible pigment that fluoresced when exposed to ultra-violet irradiation, resembling a type of fluorescein, or pyoverdine. Pyoverdines (PVDs) are water-soluble fluorescent pigments produced by many different Pseudomonads 37. Numerous genes involved in PVD synthesis have been found within *Pseudomonas* species and can vary considerably in their number and distribution 38. The possibility that this pigment could be a type of pyoverdine should be studied in future research on this *Pseudomonas* species.

### Other implications

The genomes of* P. carnis* UCD_MED3 and *P. carnis* UCD_MED7 are nearly identical (ANI= 99.9968%) suggesting the strains may have initially come from the same source within the home refrigerator where the soy products were stored. However, tracking the source of contamination was not possible.

Pigment production may be enhanced by the high amino acid content of soybeans since amino acids can be utilized for production of secondary metabolites such as pigments when bacteria are confronted with stressful environmental conditions such as cold temperatures. The discovery of the ability of *P. carnis* UCD_MED3 and UCD_MED7 to cause discoloration in partially used soy products is an important observation because the contaminants are not likely to be detected by quality control assays or shelf-life screening if the samples chosen are unopened and not yet exposed to air 8. The blue-grey color generating ability of aerobic *Pseudomonas* species may go undetected in routine food shelf-life testing by quality control workers, because its presence might only be noticed after the package has been opened and exposed to air, by consumers. Thus, the operators of food processing facilities should maintain routine microbial testing even if contamination is not obvious or apparent because defects in these products could cause loss of sales or profits. The deposition and publication of these bacterial contaminant whole genome sequences and 16S rRNA gene sequences contribute to background information that can be used for future investigations into proper food handling and processing methods, food contamination prevention, and possibly even natural food coloring ingredients.

## Figures and Tables

**Table 1:** Isolate source, identification label, media type, and colony morphology

Source	Strain ID	Media type 1	Colony appearance on media type 1	Media type 2	Colony appearance on media type 2
tofu	UCD_MED2	LB	Light yellow	TSA	Off white, no change to agar
tofu	UCD_MED3	LB	Medium yellow, green tint	TSA	Brown, dark brown agar
soymilk	UCD_MED5	R2A	larger, white- off white	TSA	Off white, no change to agar
soymilk	UCD_MED7	LB	Medium yellow, green tint	TSA	Dark brown, dark brown agar

**Table 2:** Visual observations of soymilk inoculates after incubation at room temperature and after incubation at 4 °C. Growth was not observed in the uninoculated soymilk (negative control)

Soymilk inoculation	48h (RT)	4d (4 °C)
UCD_MED5	White, white biofilm	White, white biofilm
UCD_MED7	Grey, black biofilm	Blue, black biofilm
UCD_MED5 + UCD_MED7	White, white biofilm	White, purple biofilm
(-) Soymilk	No growth	No growth

**Table 3:** 16S rRNA gene sequences and most significant BLASTn matches

Sample ID	% Identity	Query cover	Highest sequence identity (NCBI)
UCD_MED2	99.87	100%	*Serratia liquefaciens* strain FG3
			GCF_006970665.1
UCD_MED3	99.87	100%	*Pseudomonas* sp. strain J380
			GCF_009827115.1
UCD_MED5	99.80	100%	*Serratia liquefacien*s strain S1
			GCF_008364325.2
UCD_MED7	99.87	100%	*Pseudomonas* sp. strain J380
			GCF_009827115.1

**Table 4:** Genome sequencing reads and assembly details for UCD_MED3 and UCD_MED7 as obtained from MicrobesNG

*Trimmed reads:*					
**Sample ID**	**# of reads**	**Mean coverage**	**Mean coverage excluding 0s**	**# of reads w/ insert size >300**	
UCD_MED3	1261370	95.1432	95.1618	1025286	
UCD_MED7	1750514	131.347	131.359	1405607	
					
*Assembly*:					
**Sample ID**	**# contigs**	**Largest contig**	**Total contig length**	**GC (%)**	**N50**
UCD_MED3	88	462739	6295231	59.89	149513
UCD_MED7	82	462739	6294075	59.89	176388

**Table 5:**
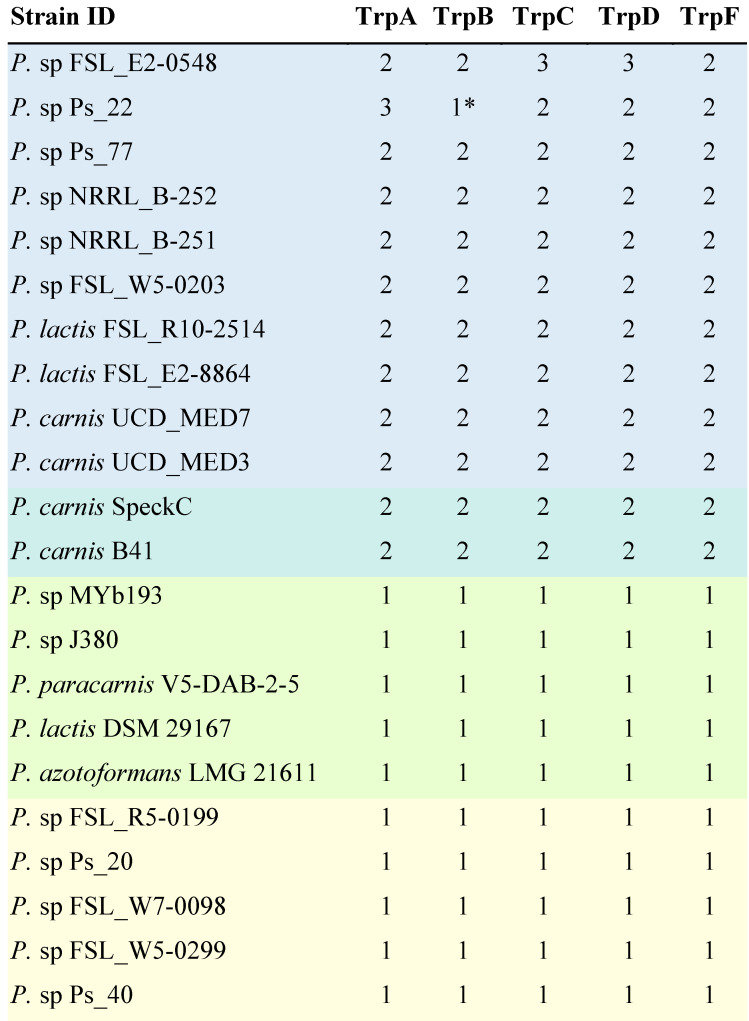
The presence of multiple homologs of tryptophan biosynthesis genes in a genome is associated with blue pigment production

*Pseudomonas* strains that have been confirmed to produce blue pigment are highlighted in blue. Strains with two homologs of each *trp* gene but which have not been reported to produce blue pigment are highlighted in blue-green. Strains which are not reported to produce a blue pigment and have only one homolog of each gene are highlighted in green. Strains for which blue pigment production was tested but was not observed and contain only one gene are highlighted in yellow. The number of homologs per genome was determined by protein level searches against the annotated proteome of each isolate using BLASTp searches with query proteins from *Pseudomonas* strain Ps_77 and using an E-value cutoff of 10×E^-10^. * Indicates that a second homolog is probably present 24.

**Table 6:** BLASTp search showing the number of homologous proteins associated with pyomelanin production within each genome

Strain ID	PhhA	AspC	HppD	TyrB	PhhB
*P. carnis* UCD_MED3	1	2	2	2	1
*P. carnis* UCD_MED7	1	2	2	2	1

The number of homologs per genome was determined by protein level searches against the annotated proteome of each isolate using BLASTp searches with query proteins from *Aeromonas media* strain WS and using an E-value cutoff of 10×E^-10^.
